# Multiple osteochondromas

**DOI:** 10.1186/1750-1172-3-3

**Published:** 2008-02-13

**Authors:** Judith VMG Bovée

**Affiliations:** 1 Department of Pathology, Leiden University Medical Center, Leiden, The Netherlands

## Abstract

Multiple osteochondromas (MO) is characterised by development of two or more cartilage capped bony outgrowths (osteochondromas) of the long bones. The prevalence is estimated at 1:50,000, and it seems to be higher in males (male-to-female ratio 1.5:1). Osteochondromas develop and increase in size in the first decade of life, ceasing to grow when the growth plates close at puberty. They are pedunculated or sessile (broad base) and can vary widely in size. The number of osteochondromas may vary significantly within and between families, the mean number of locations is 15–18. The majority are asymptomatic and located in bones that develop from cartilage, especially the long bones of the extremities, predominantly around the knee. The facial bones are not affected. Osteochondromas may cause pain, functional problems and deformities, especially of the forearm, that may be reason for surgical removal. The most important complication is malignant transformation of osteochondroma towards secondary peripheral chondrosarcoma, which is estimated to occur in 0.5–5%. MO is an autosomal dominant disorder and is genetically heterogeneous. In almost 90% of MO patients germline mutations in the tumour suppressor genes *EXT1 *or *EXT2 *are found. The *EXT *genes encode glycosyltransferases, catalyzing heparan sulphate polymerization. The diagnosis is based on radiological and clinical documentation, supplemented with, if available, histological evaluation of osteochondromas. If the exact mutation is known antenatal diagnosis is technically possible. MO should be distinguished from metachondromatosis, dysplasia epiphysealis hemimelica and Ollier disease. Osteochondromas are benign lesions and do not affect life expectancy. Management includes removal of osteochondromas when they give complaints. Removed osteochondromas should be examined for malignant transformation towards secondary peripheral chondrosarcoma. Patients should be well instructed and regular follow-up for early detection of malignancy seems justified. For secondary peripheral chondrosarcoma, *en-bloc *resection of the lesion and its pseudocapsule with tumour-free margins, preferably in a bone tumour referral centre, should be performed.

## Disease name and synonyms

Multiple Osteochondromas (MO) MIM 133700

Hereditary Multiple Exostoses (HME), Multiple Hereditary Exostoses (MHE), EXT, diaphyseal aclasis, (multiple hereditary) osteochondromatosis, multiple cartilaginous exostoses

## Definition and diagnostic criteria

Osteochondroma (osteocartilaginous exostosis) is a cartilage capped bony projection arising on the external surface of bone containing a marrow cavity that is continuous with that of the underlying bone [1]. A diagnosis of MO can be made when radiologically at least two osteochondromas of the juxta-epiphyseal region of long bones are observed. In the majority of patients a positive family history and/or mutation in one of the *EXT *genes can be detected [2,3].

## Epidemiology

The prevalence of MO is estimated at 1:50,000 persons within the general population [4] and seems to be higher in males (male-to-female ratio 1.5:1) [2,5]. This is probably due to the fact that females tend to have a milder phenotype and are therefore more easily overlooked [2]. The solitary (sporadic) form of osteochondroma is approximately six times more common than the occurrence within the context of MO. Approximately 62% of the patients with multiple osteochondromas have a positive family history [2].

## Clinical description

**Figure 1:**
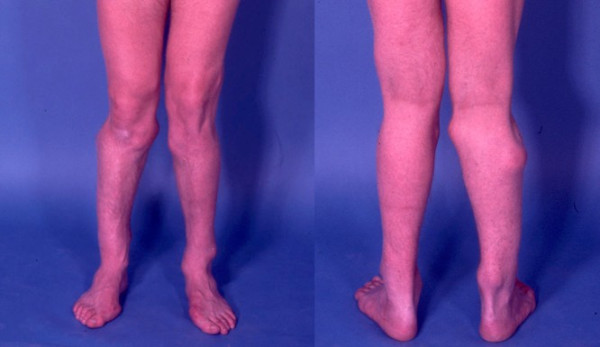
Photograph of the legs of a 26 year old male showing multiple lumps leading to deformity.

**Figure 2:**
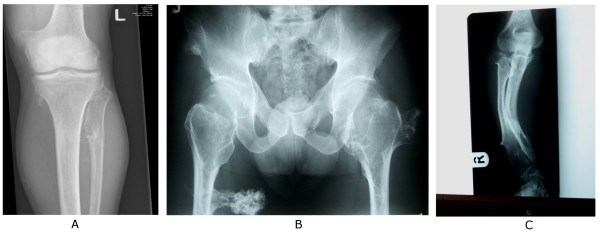
Examples of radiographs demonstrating multiple osteochondromas around the knee (A) and at the pelvis and proximal femur (B), while (C) demonstrates the deformity of the forearm (shortening of the ulna with secondary bowing of radius) that is found in 39–60% of the patients.

Osteochondromas develop and increase in size in the first decade of life, ceasing to grow when the growth plates close at puberty. They are pedunculated or sessile (broad base) and can vary widely in size. The majority are asymptomatic and located in bones that develop from cartilage, especially the long bones of the extremities, predominantly around the knee (Figures 1 and 2A). The facial bones are not affected. The number of osteochondromas may vary significantly within and between families, the mean number of locations is 15–18 [6]. In addition, in MO patients a variety of orthopaedic deformities can be found like deformities of the forearm (shortening of the ulna with secondary bowing of radius) (39–60%) [4,6,7] (Figure 2C), inequality in limb length (10–50%) [4,7], varus or valgus angulation of the knee (8–33%) [4,7], deformity of the ankle (2–54%) [4,7] and disproportionate short stature (37–44%) [2,5,6].

Other complications of the osteochondromas include osseous and cosmetic deformities, bursa formation, arthritis (14%) [5] and impingement on adjacent tendons, nerves (22.6%) [5], vessels (11.3%) [5] or spinal cord (0.6%) [5,8]. MO patients may have abnormal scar formation [9]. Osteochondromas bear the risk for fracture of the bony stalk during physical exercise. This is estimated to occur in approximately 5% of osteochondromas [10] and may be reason for surgical removal.

The majority of MO patients experiences pain [11,12], approximately half of which concerns generalised pain [11]. Therefore, the number of MO individuals having pain has been underestimated and pain seems a problem that must be addressed when caring for MO patients. The occurrence of pain was associated with MO related complications and surgery [11].

The most important complication of MO is malignant transformation of an osteochondroma, which is estimated to occur in 0.5–5% of patients [2,4,5,13,14]. Clinical signs of malignant transformation include an increase in size and pain [6]. Malignant transformation of osteochondroma leads to a secondary peripheral chondrosarcoma in 94% of the cases [15]. The suspicion of secondary chondrosarcoma is indicated by growth of the tumour after puberty, the presence of pain, or a thickness over 1 cm of the cartilaginous cap in adults.

## Aetiology

Two genes, *EXT1 *and *EXT2 *located respectively at 8q24 and 11p11-p12, have been isolated to cause MO [16-19]. Additional linkage to chromosome 19p has been found, suggesting the existence of an *EXT3*-gene [20]. However, the gene has never been identified. Moreover, the increased sensitivity of mutation detection and the use of new techniques screening for larger deletions, such as MLPA, have dramatically decreased the proportion of MO patients without an *EXT1 *or *EXT2 *mutation to <15% [21-23]. These data question the existence of an *EXT3*-gene at 19p.

The *EXT1 *gene is composed of 11 exons and has a coding region of 2238 bp [17-24]. The *EXT2*-gene contains 16 exons [18,19] and its cDNA defines a single open reading frame of 2154 bp. EXT1 and EXT2 are highly similar, especially in the carboxy terminal region [18,19].

The *EXT1 *gene was reported to show linkage in 44%–66% of the MO families [25,26], whereas *EXT2 *would be involved in 27% [26]. Germline mutations of *EXT1 *and *EXT2 *in MO patients have been studied extensively in Caucasian as well as Asian populations [27]. In *EXT1*, mutations are more or less randomly distributed over the first 6 exons, while the last 5 exons, containing the conserved carboxyterminal region, contain significantly less mutations [27]. Similarly, in *EXT2 *most mutations are found in the first eight exons. No mutational hotspots are found. Approximately 80% of the mutations are either non-sense, frameshift, or splice-site mutations leading to premature termination of EXT proteins [25,28-32]. The majority of missense mutations also lead to defective EXT protein function [33]. Mutations in *EXT1 *seem associated with a more severe phenotype as compared to *EXT2 *[34-37].

It has long been thought that osteochondromas are the result of skeletal dysplasia. It is now however generally accepted that osteochondromas are neoplastic, since genetic changes are found in the cartilage cap [1,38-42]. The *EXT*-genes are tumour suppressor genes. Loss of the remaining *EXT1 *wildtype allele has been demonstrated in the cartilage cap of osteochondromas from MO patients [39]. However, in a considerable proportion of MO patients loss of the remaining wildtype allele could not be detected so far [43]. In seven out of eight solitary osteochondromas, homozygous deletions of *EXT1 *are found [38] further supporting the two-hit model. Moreover, the deletions were confined to the cartilage cap. Thus, the cartilage cap is the clonal neoplastic element, while the stalk is reactive [38].

Both *EXT1 *and *EXT2 *mRNA is ubiquitously expressed [17-19]. A high level of expression of Ext1 and Ext2 mRNA has been found in developing limb buds of mouse embryos [44,45] and expression was demonstrated to be confined to the proliferating and prehypertrophic chondrocytes of the growth plate [46]. In osteochondromas and peripheral chondrosarcomas the expression of *EXT1 *and/or *EXT2 *is decreased, corresponding to the mutation status [47].

The gene products, exostosin-1 (EXT1) and exostosin-2 (EXT2), are endoplasmic reticulum localized type II transmembrane glycoproteins which form a Golgi-localised hetero-oligomeric complex that catalyzes heparan sulphate (HS) polymerization [48-51]. Heparan sulphate proteoglycans (HSPG) are large macromolecules composed of heparan sulphate glycosaminoglycan chains linked to a protein core. Four important HSPG families are syndecan, glypican, perlecan and isoforms of CD44 bearing variable exon 3 (CD44v3). In osteochondromas in which EXT expression is decreased due to mutation or deletion, the heparan sulphate proteoglycans seem to accumulate in the cytoplasm of the cell, instead of being transported to be expressed at the cell surface [47].

**Figure 3:**
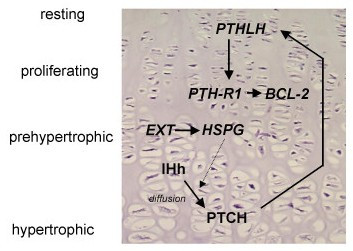
**Growth plate signaling in the normal growth plate.** Indian Hedgehog protein (IHh) is expressed in the prehypertrophic cells, and diffuses over a variable distance to its receptor Patched (PTCH). Subsequently, increased secretion of ParaThyroid Hormone Like Hormone (PTHLH) is induced at the apical perichondrium via an incompletely understood mechanism. PTHLH then diffuses to its receptor, whose expression is restricted to the late proliferating chondrocytes, inhibiting their further differentiation, resulting in less IHh producing cells, which closes the feedback loop. Thus, PTHLH regulates the pace of chondrocyte differentiation by delaying the progression of chondrocytes towards the hypertrophic zone, allowing longitudinal bone growth. Defective or absent EXT proteins leading to altered or absent HSPG expression at the cell surface may affect this negative feedback loop by disturbing the diffusion of IHh, produced at the pre-hypertrophic chondrocytes, towards its receptor Ptc.

EXT and HSPGs are required for high-affinity binding of fibroblast growth factor to its receptor and for the diffusion of the morphogens Hedgehog (Hh, human homologues Indian (IHH) and Sonic Hedgehog (SHH) [52-54], decapentaplegic (dpp, human homologues TGF-beta and BMP) and wingless (wng, human homologue Wnt) [55,56]. These three pathways are important during development and are specifically active in the growth plate during endochondral bone formation. During normal growth, IHh and PTHLH are involved in a delicate paracrine feedback loop regulating proliferation and differentiation of the chondrocytes of the growth plate (Figure 3). In osteochondroma, IHH signalling is still active and is probably cell autonomous [57,58]. PTHLH signalling, which is downstream of IHH and is responsible for chondrocyte proliferation, is absent in osteochondroma, while being upregulated upon malignant transformation of osteochondroma [59,60]. Wnt signalling and TGF-beta signalling are also active in the majority of osteochondromas [57]. The exact role of EXT in orchestrating these pathways leading to osteochondroma formation in MO patients needs to be further elucidated.

## Diagnostic methods

When a patient is suspected to have MO, the full radiological documentation, histology (if available), patient history and family history have to be carefully reviewed. Given the specific radiological and histological expertise needed, and the rarity of the disorder and of those in the differential diagnosis, it is recommended that this review is performed by specialists in the field, for instance through a national bone tumour registry consisting of clinicians, radiologists and pathologists. If this review is indicative for MO, the peripheral blood of the patient may be screened for germline mutations in *EXT1 *or *EXT2 *[61]. In case of a positive family history in which MO is clearly established in relatives, the diagnosis of MO can be clinically made and mutation analysis is not essential. With the currently used methods it is possible to detect point mutations or gross deletions in almost 90% of MO patients [21-23,61-63].

To evaluate possible malignant transformation in case of complaints or growth of the lesion after puberty, the size of the cartilaginous cap can be well established with T2-weighted magnetic resonance (MR) imaging [64]. A cartilage cap >1.5 cm should be regarded with caution. The role of 18 Fluoro-deoxyglucose positron emission tomography (18FDG PET) needs to be further established [65].

## Differential diagnosis

Dysplasia Epiphysealis Hemimelica (DEH, Trevor's disease, tarso-epiphysial aclasis) and metachondromatosis (MC) are considered in the differential diagnosis of solitary and hereditary osteochondromas. Despite their similarities, they were shown to be separate entities [66] and the EXT downstream pathway is not involved [67].

DEH is a developmental disorder with cartilaginous overgrowth of a portion of one or more epiphyses [68]. It predominantly affects the lower extremity on one side of the body. It is usually restricted to either the medial (most frequent) or lateral side of the limb (hemimelic). Similar to osteochondroma, DEH is usually diagnosed prior to the age of 15 years, more often in boys than in girls, and growth of these lesions end at puberty as the growth plates close [68,69]. In contrast to MO, malignant transformation has not been reported so far [68] and there does not appear to be any genetic transmission [69-71].

MC is a rare disorder exhibiting, synchronous, both multiple osteochondromas and enchondromas in children. It has an autosomal dominant mode of inheritance [72-74] but the disorder has not been mapped in the human genome so far. MC related osteochondromas characteristically occur in the hands and feet, predominantly the digits and toes, and point toward the adjacent growth plate, while in MO the osteochondromas are mainly located in the long or other tubular bones and point away from the epiphysis [72]. Differentiation from MO is of great clinical significance because in patients with MC the lesions do not result in shortening or deformity of affected bones as in MO, and may spontaneously decrease in size or resolve completely, both clinically and radiologically [72,74].

Moreover, MO should be distinguished from enchondromatosis (Ollier disease and Maffucci syndrome), in which multiple cartilage tumours are found in the medulla of bone, with a predilection for the short tubular bones and a unilateral predominance [75].

Upon histopathological examination of osteochondroma after surgical removal malignancy should be considered. Malignant transformation in the cartilage cap of osteochondroma leads to a secondary peripheral chondrosarcoma. Occasionally, osteosarcomas and spindle cell sarcomas develop in the stalk of the osteochondroma [15,76-80]. Extremely rare is the occurrence of dedifferentiated peripheral chondrosarcoma, in which a low-grade chondrosarcoma that developed within an osteochondroma "dedifferentiates" into a high grade sarcoma [81,82].

## Genetic counselling

MO is an autosomal dominant disorder. Affected individuals have 50% risk of transmitting the disorder to their offspring. MO has nearly 100% penetrance. If the exact mutation is known antenatal diagnosis is technically possible.

## Management including treatment

Osteochondromas are only removed when they cause pain, when they give functional complaints for instance due to compression on nerves or vessels, or for cosmetic reasons.

Surgical treatment of forearm deformities remains controversial. In a retrospective series 23 MO patients corrective osteotomy and/or lengthening of forearm bones was not beneficial [83]. Moreover, one should consider the possible recurrence of ulnar shortening within 1.5 years when operating skeletally immature patients [83,84]. The most beneficial procedure was excision of the osteochondromas. The simple removal of an osteochondroma can improve forearm rotation and correct deformity [83], especially if there is an isolated tumour of the distal part of the ulna.

If the diagnosis of MO is established and all tumours are identified, patients should be well instructed to seek earlier medical attention if their condition changes, for instance if there is pain or growth of a known lesion [61]. It is important to realise that no new osteochondromas develop after puberty. Moreover, regular follow-up to discover potential malignant transformation at an early stage to enable adequate treatment should be considered. The risk of malignant transformation of osteochondroma towards secondary peripheral chondrosarcoma is estimated at 1–5% [2,4,5,13,14,34]. After skeletal maturation a base-line bone scan is recommended [61]. Furthermore, baseline plain radiographs of areas that can not be manually examined, like the chest, pelvis and scapula can be performed [61]. After the base-line documentation one should consider screening patients regularly, for instance every year or every other year. There are as yet no studies available that have proven efficacy of screening. If lesions change over time, further examination, using magnetic resonance (MR) imaging including contrast enhanced MR sequences, is indicated [61].

In case of malignancy, *en-bloc *resection of the lesion and its pseudocapsule with tumour-free margins, preferably in a bone tumour referral centre, should be performed, resulting in excellent long term clinical and local results. The most common location is however the pelvis where the large cartilage cap can be difficult to excise. In a series of 61 patients with grade I or II secondary peripheral chondrosarcoma of the pelvis published by Donati *et al*., a 3% local recurrence rate was found after wide resection, in contrast with 23% after inadequate excision [85].

## Prognosis

Osteochondromas are benign lesions and do not affect life expectancy. The risk of malignant transformation is 1–5%. The prognosis for secondary peripheral chondrosarcoma is depending on histological grade: 10 year survival rates are 83% for grade I chondrosarcomas compared to 29% for grade III chondrosarcomas [86].

## Unresolved questions

• How can the enormous difference in disease severity within and between families be explained?

• What drives malignant transformation of osteochondroma and can this be prevented?

• What is the role of EXT in normal cartilage growth and differentiation and in osteochondroma formation?
